# Utilization of integrated community-based case management of childhood illness and associated factors in Ethiopia: a systematic review and meta-analysis

**DOI:** 10.1186/s13052-024-01702-0

**Published:** 2024-07-30

**Authors:** Maru Mekie, Yismaw Yimam Belachew, Setegn Muche Fenta, Wassie Yazie Ferede, Enyew Dagnew Yehuala, Wubet Taklual, Demewoz Kefale Mekonen, Dagne Addisu

**Affiliations:** 1 College of Health Sciences, Department of Midwifery, Debre Tabor University, PO.BOX: 272, Debre Tabor, Ethiopia; 2 College of Health Sciences, School of Medicine, Department of Gynecology and Obstetrics, Debre Tabor University, Debre Tabor, Ethiopia; 3 College of Natural and Computational Sciences, Department of Statistics, Debre Tabor University, Debre Tabor, Ethiopia; 4 College of Health Sciences, School of Public Health, Debre Tabor University, Debre Tabor, Ethiopia; 5 College of Health Sciences, Department of Pediatrics and Child Health Nursing, Debre Tabor University, Debre Tabor, Ethiopia

**Keywords:** ICCM, Utilization, Associated factors, Child mortality, Ethiopia

## Abstract

**Background:**

Despite significant progress being made in reducing under-five mortality, three-fourths of under-five deaths are still caused by preventable conditions such as pneumonia, diarrhea, malaria, and newborn issues. Integrated community case management of childhood illnesses (ICCM) could serve as a means to reduce preventable child mortality in Low- and Middle-Income countries. Our aim was to assess the overall level of ICCM utilization and its associated factors in Ethiopia.

**Methods:**

Candidate studies for inclusion in this review were identified through searches across various databases, including PubMed, EMBASE, Google Scholar, and university repositories online databases, spanning from February 1, 2024, to March 18, 2024. The quality assessment of the studies included in this systematic review and meta-analysis was conducted using the Newcastle-Ottawa Quality Assessment Scale (NOS). Data extraction and analysis were carried out using Microsoft Excel and Stata 17 software, respectively. Heterogeneity among the studies was assessed using Cochran’s Q test and I^2^ statistics, while the presence of publication bias was evaluated through funnel plots and Egger’s regression asymmetry test. Subgroup analysis was performed based on sample size and study site.

**Results:**

In this study, the pooled level of ICCM utilization was found to be 42.73 (95%, CI 27.65%, 57.80%) based on the evidence obtained from ten primary studies. In this review, parents’ awareness about illness (OR = 2.77, 95%, CI 2.06, 3.74), awareness about ICCM service (OR = 3.64, 95%, CI 2.16, 6.14), perceived severity of the disease (OR = 3.14, 95%, CI 2.33, 4.23), secondary/above level of education (OR = 2.57, 95%, CI 1.39, 4.77), and live within 30 min distance to the health post (OR = 3.93, 95%, CI 2.30, 6.74) were variables significantly associated with utilization of ICCM in Ethiopia.

**Conclusion:**

The utilization of ICCM was found to be low in Ethiopia. Factors such as parents’ awareness about the illness, knowledge of ICCM services, perceived severity of the disease, attending a secondary or more level of education, and living within 30 min distance to the health post were significantly associated with the utilization of ICCM. Therefore, it is crucial to focus on creating awareness and improving access to high-quality ICCM services to reduce child morbidity and mortality from preventable causes.

**Supplementary Information:**

The online version contains supplementary material available at 10.1186/s13052-024-01702-0.

## Background

Integrated Community Case Management (ICCM) is a strategy to train, support, and supply community health workers (CHW) to provide diagnostic, treatment, and referral services for common, treatable, and curable childhood illnesses such as malaria, pneumonia, and diarrhea. ICCM brings the diagnosis and management of childhood illnesses closer to individual homes [1].

ICCM is a strategy to increase access to effective case management for young children suffering from malaria, pneumonia, and diarrhea in areas where access to health services is limited. The current evidence indicates that ICCM is a key public health strategy to increase coverage of quality treatment services for children, especially in malaria-endemic countries in Africa [2]. ICCM can also increase early care seeking for illness and early access to appropriate treatment for children which decreases morbidity and mortality for children under five years of age [2].

The sustainable development Goal aimed to end preventable deaths of newborns and children under five years of age with all countries aiming to reduce neonatal mortality to as low as 12 per 1000 and under five years of mortality as low as 25 per 1000 live births [3] which could be far from success for most low and middle-income countries.

Globally, remarkable progress in child survival intervention has been made and millions of children have better survival chances than in 1990. The global under-five mortality rate fell to 39 per 1000 live births in 2017. Despite the progress made in reducing under-five mortality, 75% of under-five deaths are still caused by pneumonia, diarrhea, malaria, and newborn conditions [1, 4]. According to a study published in Lancet Health, 49.2% of under-five mortality was due to infectious causes of which 13.9% and 9.1% of deaths were attributable to lower respiratory infections and diarrheal diseases respectively. According to the study, the majority of under-five deaths can be prevented by low-cost intervention [5]. ICCM could be the most feasible intervention to prevent under-five morbidity and mortality from preventable causes in low- and middle-income countries including Ethiopia.

In Ethiopia, under-five mortality is almost twice that of the global burden, 67 per 1000 live births based on the evidence obtained from the Ethiopia Demographic and Health Survey (EDHS) [6]. Over two-thirds of childhood deaths in Ethiopia are caused by few and easily preventable conditions; mainly infections, neonatal conditions, and malnutrition [7]. There is a long way to go to achieve the sustainable development goal in terms of neonatal, infant, and child mortality in the country [3]. The Ministry of Health of Ethiopia has implemented various interventions, including community management of childhood illnesses such as pneumonia, diarrhea, malaria, and malnutrition (ICCM), as well as neonatal sepsis (CBNC), community-based nutrition (CBN), and Newborn Corner initiatives, in order to reduce child morbidity and mortality in the country [7, 8].

Despite such interventions, there is significant child mortality attributed to preventable causes of childhood illnesses due to low utilization of available interventions [6, 7]. In many settings, with a high burden of child mortality, access to timely treatment and care is limited. Most children who die from malaria, pneumonia, or diarrhea live in areas underserved by the health system, with poor access to health facilities. To reduce preventable child mortality, improving the utilization of community-based health interventions by avoiding possible barriers is imperative [9, 10].

ICCM serves as a vital intervention strategy targeting the health needs of under-five children in vulnerable and marginalized populations. It aims to bring healthcare services closer to those in greatest need, alleviating strain on overwhelmed health systems and ensuring equitable access to quality care. In Ethiopia, ICCM is carried out by Health Extension Workers (female trained health workers; led and employed by the government of Ethiopia), who are primarily focused on enhancing the health of women and children in rural areas with limited access to healthcare services [11–13].

As per the knowledge of the authors concerned, there is no comprehensive national evidence regarding the level of ICCM utilization in Ethiopia. Different individual studies conducted in different corners of the country indicated that the level of utilization of ICCM ranges from 10.43 to 70.21% with high discrepancy [14–23]. Hence, this study aimed to determine the level of ICCM utilization and its determinant factors in the country using the available primary studies conducted in different parts of the country.

## Methods

We conducted this systematic review and meta-analysis to assess the level of integrated community case management of childhood illnesses and its determinant factors in Ethiopia through the utilization of both published and unpublished studies accessed through different databases including University repositories (Master thesis and Ph.D. Dissertations).

### Search strategy and study selection

**Fig. 1:**
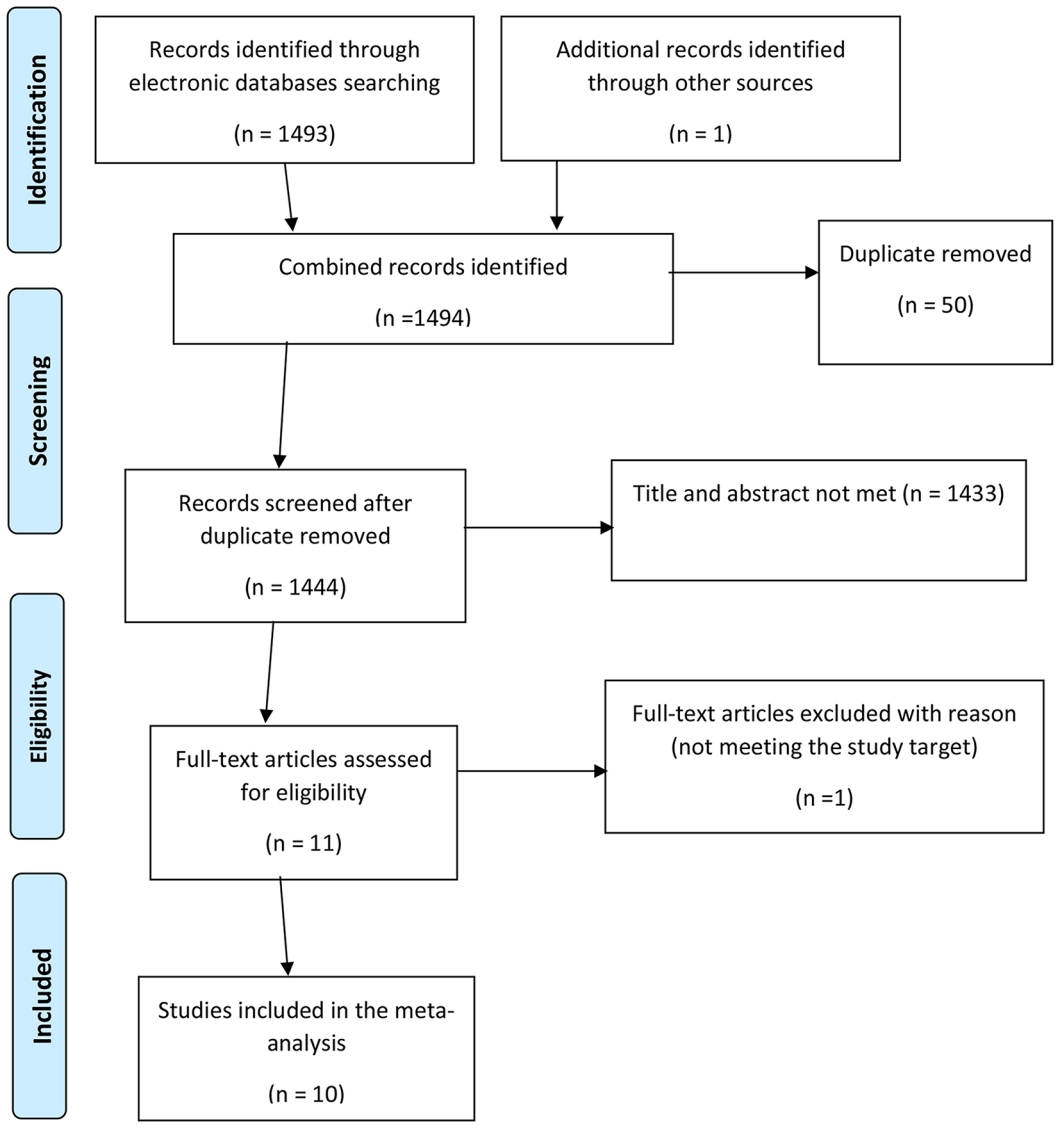
PRISMA flow chart revealing study selection for systematic review and meta-analysis of Integrated community case management of childhood illnesses in Ethiopia

Both published and unpublished studies conducted about the utilization of integrated community case management of childhood illnesses in Ethiopia were searched using different databases such as PubMed, EMBASE, Google Scholar, and University online repositories to conduct this systematic review and meta-analysis (Table 1). We have used the Preferred Reporting Items for Systematic Reviews and Meta-analyses (PRISMA) guidelines for the selection and exclusion of studies for this systematic review and meta-analysis (Fig. 1). Similarly, the PRISMA checklist 2020 (Supplementary file 1) was used to report the findings of this review [24].

**Table 1:** Search strategies used for PubMed and related databases

Databases	Search terms	Number of studies
PubMed/Medline	level[All Fields] AND integrated[All Fields] AND (“residence characteristics“[MeSH Terms] OR (“residence“[All Fields] AND “characteristics“[All Fields]) OR “residence characteristics“[All Fields] OR “community“[All Fields]) AND based[All Fields] AND (“organization and administration“[MeSH Terms] OR (“organization“[All Fields] AND “administration“[All Fields]) OR “organization and administration“[All Fields] OR “management“[All Fields] OR “disease management“[MeSH Terms] OR (“disease“[All Fields] AND “management“[All Fields]) OR “disease management“[All Fields]) AND “childhood“[All Fields] AND illnesses[All Fields] AND associated[All Fields] AND factors[All Fields] AND (“ethiopia“[MeSH Terms] OR “ethiopia“[All Fields])	1433
Other databases		60
Gray literature		1
Total Search		1494
Numbers candidates for inclusion		11
Excluded with reasons		1
Studies included in the analysis		10

### Inclusion and exclusion criteria

#### Inclusion criteria

Studies (both published and unpublished) conducted in Ethiopia on the utilization of integrated community case management of childhood illnesses and published/reported in the English language were included in this systematic review and meta-analysis. An article search was conducted from February 1, 2024, to March 18, 2024.

#### Exclusion criteria

In this review, studies that did not report the outcome of interest and were not fully accessible were excluded.

#### Data extraction

The data extraction form for the level of ICCM utilization and its predictor variables was prepared in Microsoft Excel by the first author (MM) and last author (DA). The extraction form on the Excel workbook includes author name, publication year, study region, study site, study design, study period, sample size, prevalence, and standard error of the prevalence. Likewise, the extraction form includes the logor and standard error of the logor of possible determinant factors of ICCM utilization such as distance to the health post, education level of the parents/guardians, awareness of signs of illnesses, awareness of service availability, and perceived severity of the diseases.

#### Measurement of the outcome variable

Parents/Caregivers who sought care for their sick children from a nearby health post for common signs of childhood illnesses such as fever, cough, diarrhea, pneumonia, and malaria were reported as utilizing ICCM [21, 23].

#### Data quality assurance

The titles and abstracts of studies to be included in this systematic review and meta-analysis were reviewed by the first (MM) and the last (DA) authors independently. The third (WYF) and the fourth (EDY) authors were involved in the resolution of disagreement in the inclusion and exclusion of the studies by the first and the last authors. Following the review, articles were exported to Endnote 8 to manage duplications. The Newcastle-Ottawa Quality Assessment Scale (NOS) was used to assess the quality of studies included in this systematic review and meta-analysis study. The criteria used to assess the quality of studies for inclusion in this systematic review and meta-analysis were, the sample size, non-response rate, representativeness of the sample, the ascertainment of the risk factor, management of the confounding factors, the comparability of outcome groups, the assessment of the outcome variable, and the statistical tests Hence, the included studies were found to have a quality score of 7–9 using the NOS quality assessment scale (Table 2).

**Table 2:** Characteristics of studies reporting repeat-induced abortion in Ethiopia

Author	Publication year	Region	Study area	Study design	Sample size	Population with outcome	Prevalence	Response rate	Risk of bias
Berhanu A. et al. [14].	2020	South Ethiopia Region	Wolayita, Kindo Didaye woreda	Cross-sectional	633	66	10.43	99%	Low risk
Debel N. et al. [15].	2022	Oromia Region	West Shoa, Dandi woreda	Cross-sectional	624	325	52.08	100%	Low risk
Samuel S. et al. [16].	2021	South Ethiopia Region	Wolayita, Boloso Sore Woreda	Cross-sectional	439	111	25.28	97.8%	Low risk
Yeheyis T. et al. [17].	2021	Sidama Region	Hawassa City	Cross-sectional	366	257	34.91	70.22	Low risk
Yohannes S. et al. [18].	2021	Central Ethiopia Region	Hadiya Health facilities	Cross-sectional	574	129	22.47	100%	Low risk
Salgedo WB. et al. [19].	2020	Southwest Ethiopia Region	Dawro zone	Cross-sectional	806	475	58.93	98%	Low risk
Gorfu MB. et al. (preprint) [20].	2014	Oromia Region	Arsi, Agarfa woreda	Cross-sectional	238	127	53.36	97%	Low risk
Rikiba R. et al. [21].	2023	Sidama Region	Wonsho district	Cross-sectional	835	152	18.20	100%	Low risk
Kassa EA. at al [22].	2018	Central Ethiopia Region	Hadiya, Shashogo district	Cross-sectional	422	199	47.16	98.3%	Low risk
Bellete M. et al. [23].	2021	Addis Ababa	Nifas Silk sub-city	Croo	875	608	69.49	95.31%	Low risk

### Statistical analysis

The extracted data in the Excel workbook were exported to Stata 17 software for analysis. Funnel plot and Egger’s regression asymmetry test were used to assess publication bias. In the same manner, the utilization level of ICCM service as well as its associated factors are presented using a forest plot with a 95% confidence interval. Random variation among primary studies was assessed using Cochran (Q test) and I^2^ test [25]. Hence, an I^2^ value of 0, 25%, 50%, and 75% were interpreted as no, low, moderate, and high heterogeneity respectively. The derSimonian-Laird model was used in estimating the pooled level of ICCM utilization and its associated factors.

## Results

A total of 1494 studies were retrieved through electronic databases and University repositories. Fifty studies were excluded due to duplicates, giving 1444 studies. After the review of titles and abstracts, 1433 studies were excluded. Then one study was excluded for reason (not meeting the study target) [26]. We included ten full articles in this systematic review and meta-analysis (Fig. 1).

### Characteristics of included studies

A total of 10 studies (nine published studies and one unpublished study) with a total 5812 of study participants which assessed the level of ICCM utilization and its predictors published /reported in the English language were included in this systematic review and meta-analysis.

The quality of the included studies was assessed using the NOS quality assessment tool and studies with a quality assessment score of ≥ 7 were considered as low risk for bias in this review [27](Supplementary file 2). With regards to the regional distributions, the included studies were conducted in South Ethiopia [14, 16], Southwest Ethiopia [19], Central Ethiopia [18, 22], Oromia [15, 20], Sidama Regions [17, 21], and Addis Ababa City Administration [23] (Table 2).

### The prevalence of ICCM utilization based on individual studies

The utilization of ICCM utilization was reported to be high in the study conducted by Bellete M. et al. [23] and Salgedo WB. et al. [19] with respective prevalence of 69.49% and 58.93%. Low ICCM utilization was reported in the study by Berhanu A.et al [14] and Rikiba R. et al. [21] with a prevalence of 10.43% and 18.20% respectively.

### Meta-analysis

#### The level of integrated community case management of childhood illnesses (ICCM)

**Fig. 2:**
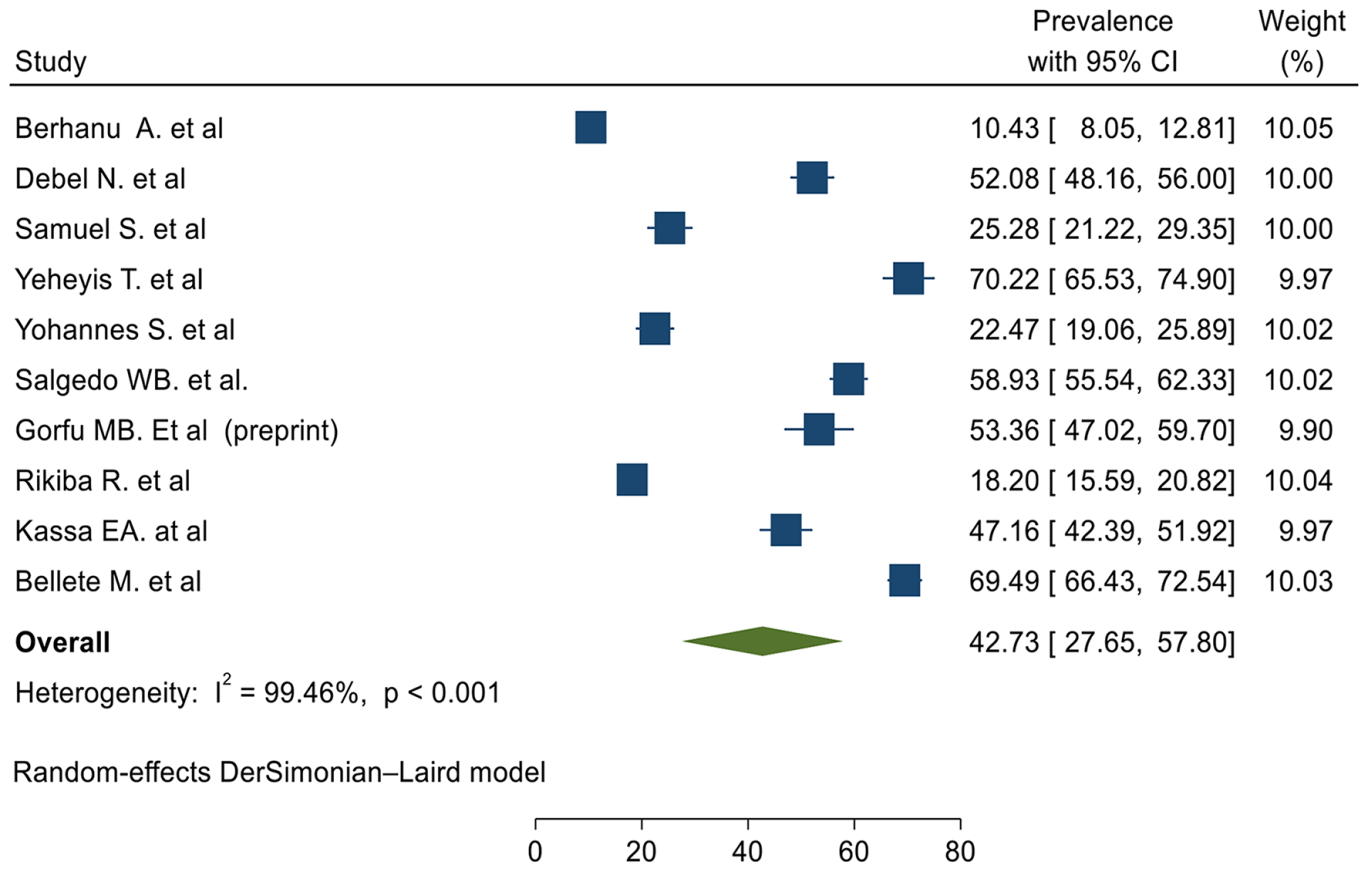
The pooled prevalence of integrated community case management of childhood illnesses in Ethiopia

Ten cross-sectional primary studies conducted in different regions of Ethiopia were used to compute the pooled level of ICCM utilization in Ethiopia [14–23]. In this study, the pooled level of ICCM utilization was found to be 42.73 (95%, CI 27.65%, 57.80%) (Fig. 2).

### Sensitivity analysis

**Fig. 3:**
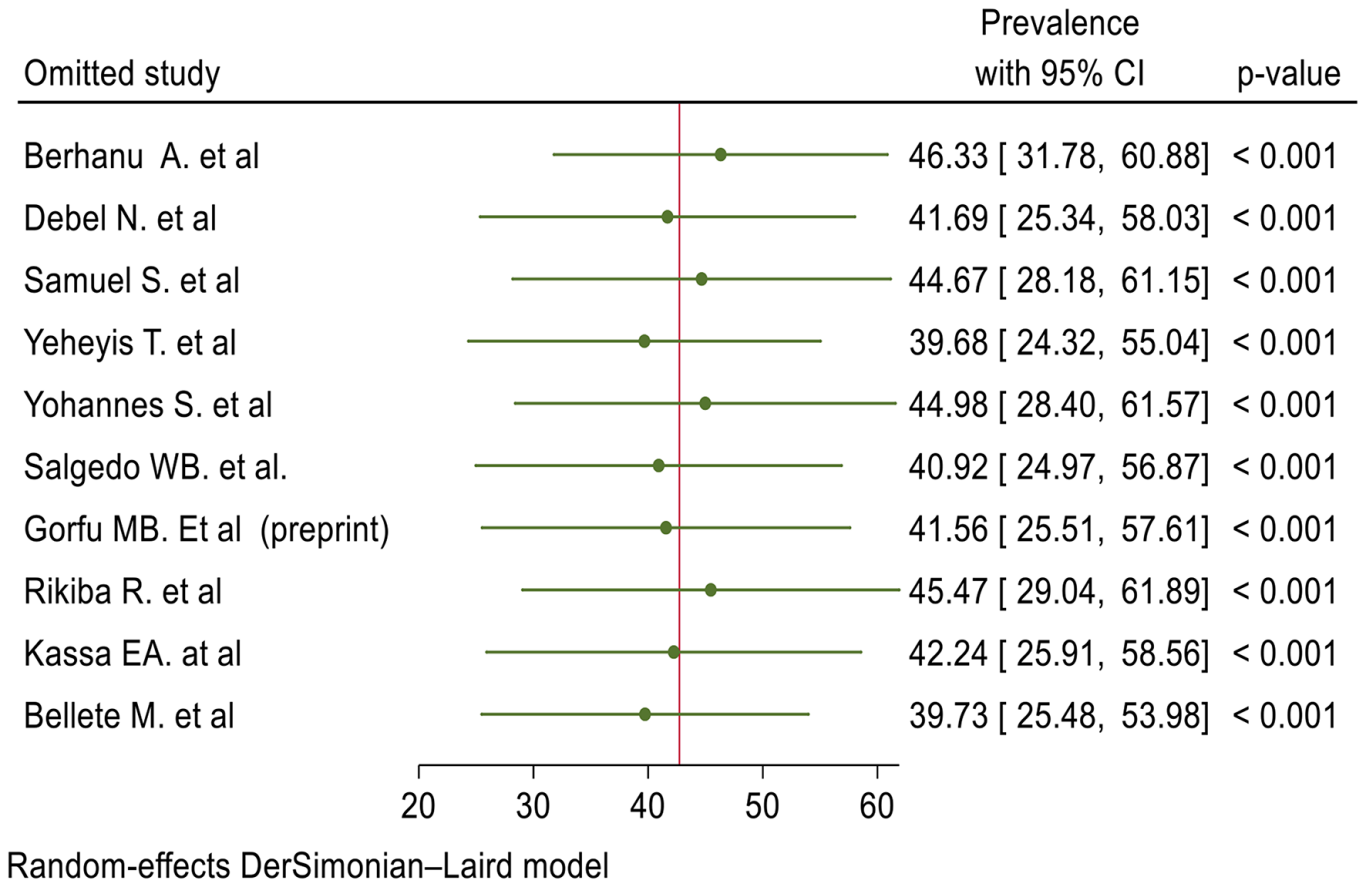
Sensitivity analysis of included studies to estimate the pooled prevalence of ICCM in Ethiopia

We conducted a sensitivity analysis to assess the influence of individual studies on the pooled prevalence of ICCM. Hence, the sensitivity analysis indicated that the point estimate of the individual study was within the confidence interval of the pooled prevalence (42.73%: 95%, CI 27.65%, 57.80%) which indicated no significant influence of individual studies (Fig. 3).

### Assessment of heterogeneity based on study site and sample size

**Fig. 4:**
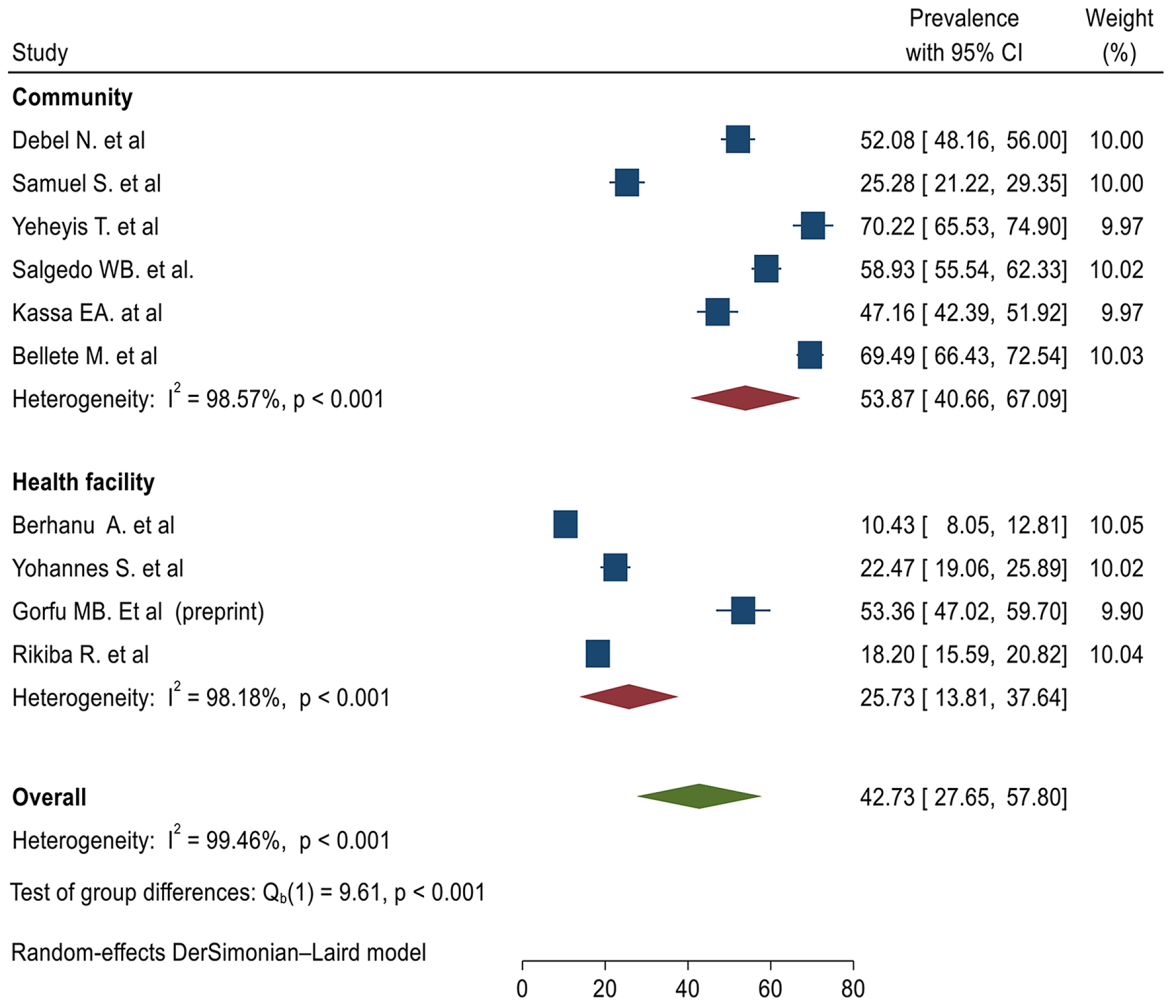
Subgroup analysis of the level of integrated community case management of childhood illnesses in Ethiopia based on the study site

**Fig. 5:**
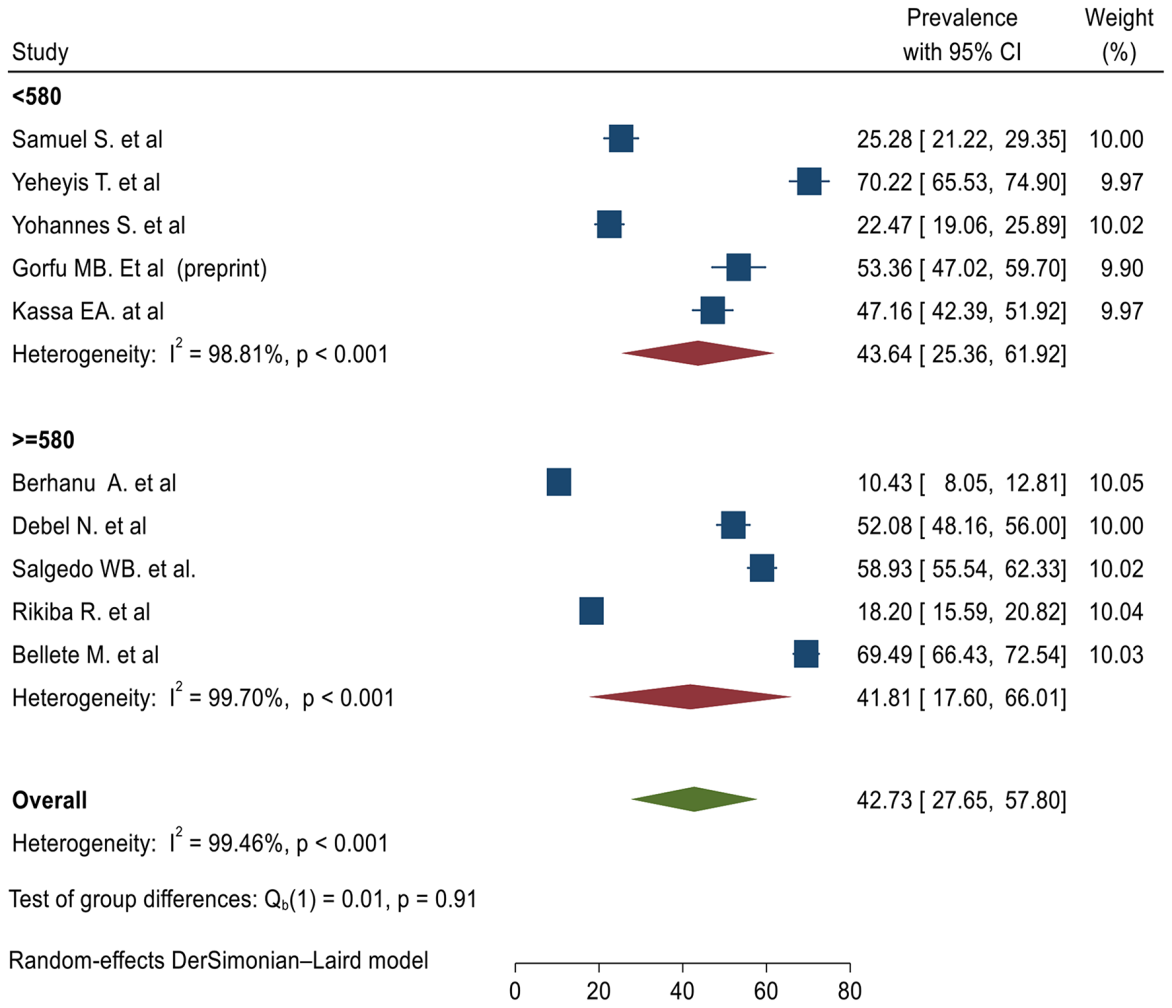
Subgroup analysis of the level of integrated community case management of childhood illnesses in Ethiopia based on sample size

High heterogeneity was observed between primary studies used to determine the pooled level of ICCM utilization (Fig. 2). Subgroup analysis based on the study site and sample size were performed to diagnose the possible cause of heterogeneity (Figs. 4 and 5).

### Subgroup analysis based on the study site

With regards to the level of ICCM utilization, there was a significant difference between study sites with a test of group difference Q _b1_ value of 9.61 and p-value of < 0.001. The level of ICCM was 53.87% (95%, CI 40.66 -67.09%) and 25.73% (95%, CI 13.81, 37.64%) among studies done at community level and facility level respectively (Fig. 4).

### Subgroup analysis based on sample size

We have used the sample mean to perform subgroup analysis based on sample size (sample size < 580 and ≥ 580). There is no significant difference in the level of ICCM between studies based on sample category with group difference test Q _b1_ value of 0.01 and p-value of 0.91. The level of ICCM utilization was found to be 43.64% (95%, CI 25.36%, 61.01%) and 41.73% (95%, CI 17.60%, 66.01%) among studies with sample size < 580 and studies with a sample size of ≥ 580 respectively (Fig. 5).

### Assessment of publication bias

**Fig. 6:**
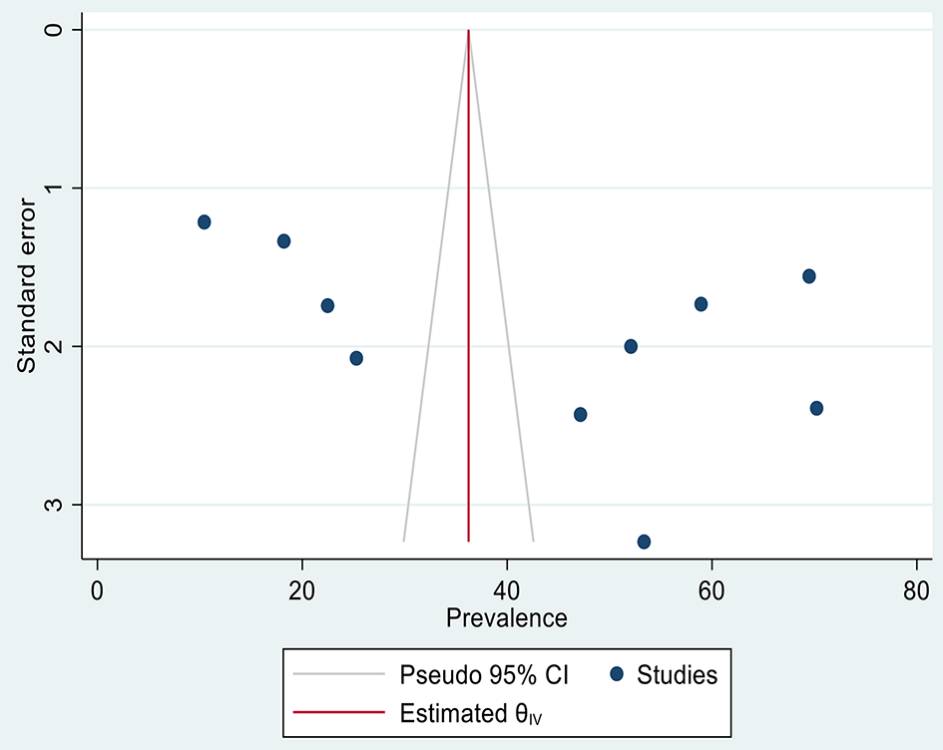
Funnel plot of included studies in a meta-analysis of ICCM utilization in Ethiopia

Funnel plot and Egger’s asymmetry test were used to assess the presence of publication bias between primary studies. The funnel plot (Fig. 6) and Egger’s asymmetry test indicated no significant publication bias with a p-value of 0.157.

### Factors associated with the utilization of ICCM in Ethiopia

**Fig. 7:**
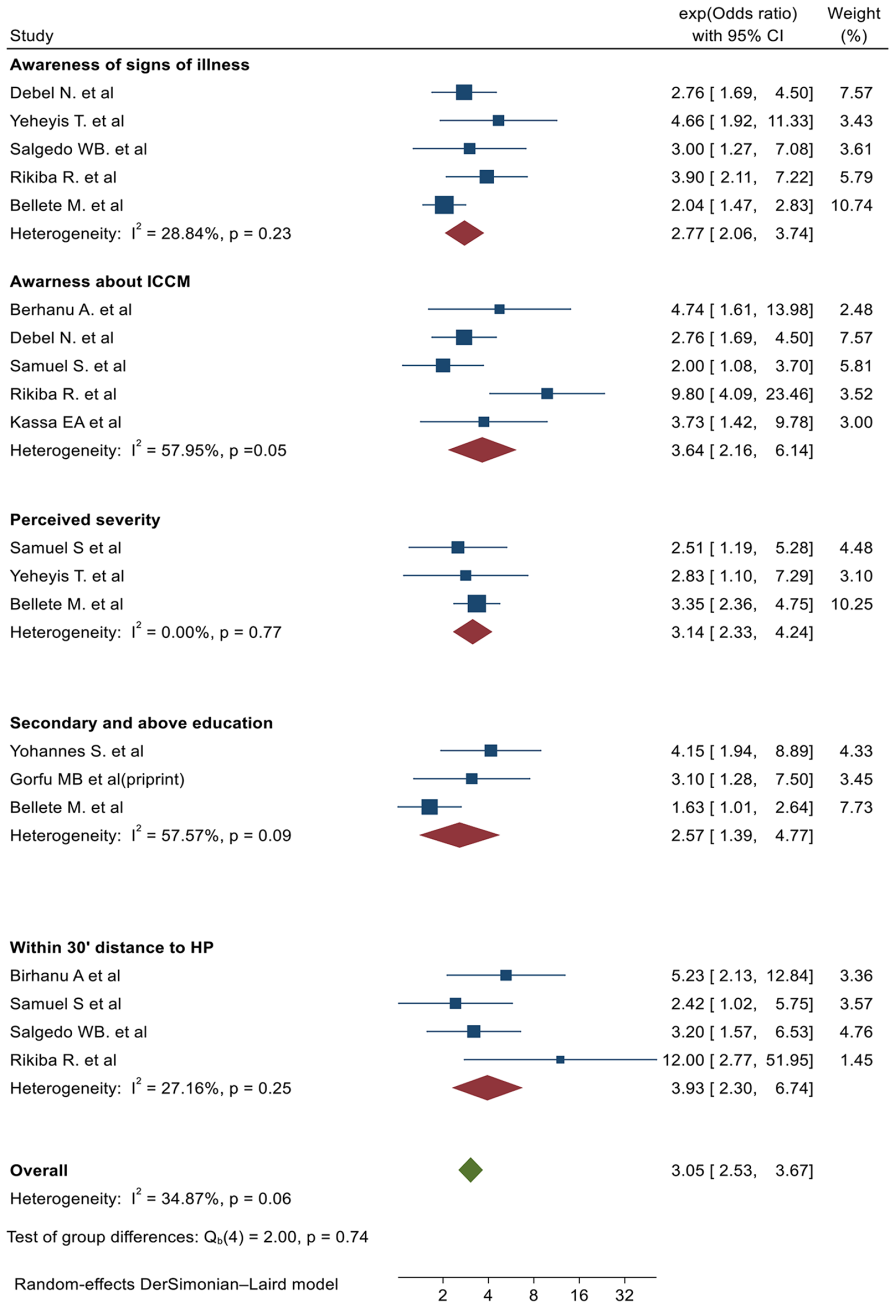
Forest plot of variables associated with utilization of ICCM in Ethiopia

In this review, parents’ awareness about the illness, awareness about ICCM service, perceived severity of the disease, secondary/above level of education, and living within 30 min distance to the health post were variables significantly associated with utilization of ICCM in Ethiopia (Fig. 7).

There was a statistically significant association between parents’ awareness of illness and utilization of ICCM. The odds of utilizing ICCM service were found to be 2.77 higher among parents who have awareness about signs of illnesses compared to counterparts (OR = 2.77, 95%, CI 2.06, 3.74). Five primary studies were used to assess the association between awareness about signs of illnesses and ICCM utilization [15, 17, 19, 21, 23] with no significant heterogeneity between studies (I^2^ = 28.84%, *P* = 0.23). In the same manner, parents who have awareness about the availability of ICCM service were 3.64 times more likely to use ICCM service [14–16, 21, 22] compared to those who do not have awareness about the availability of ICCM service with moderate heterogeneity between primary studies (OR = 3.64, 95%, CI 2.16, 6.14, (I^2^ = 57.96%, *P* = 0.05)). (Fig. 7).

Perceived severity of diseases was found to be significantly associated with ICCM utilization compared to counterparts [16, 17, 23]. The odds of ICCM utilization were found to be3.14 times higher among those who had high perceived severity about diseases compared to counterparts with no heterogeneity between primary studies (OR = 3.14, 95%, CI 2.33, 4.23), I^2^ = 0.00%, *P* = 0.77). Parents’ level of education was also found to be significantly associated with ICCM utilization based on pooled evidence from three primary studies. Parents with secondary or above level of education were 2.57 times more likely to utilize ICCM for their sick children compared to counterparts (OR = 2.57, 95%, CI 1.39, 4.77), I^2^ = 57.57%, *P* = 0.09) with minor heterogeneity between primary studies. Similarly, those who lived within 30 min distance of the health post were 3.93 times more likely to use ICCM service compared to those who live in areas more than 30 min away from a health post (OR = 3.93, 95%, CI 2.30, 6.74) with no significant heterogeneity( I^2^ = 27.16, *P* = 0.25 ) between primary studies [14, 16, 19, 21] (Fig. 7).

## Discussion

Enhancing access and utilization of ICCM services is crucial for reducing preventable child mortality in low- and middle-income countries such as Ethiopia, where access to health services is constrained. This review examines the overall level of ICCM utilization and its associated factors in Ethiopia, drawing from primary observational studies conducted across various regions of the country. The overall pooled level of ICCM utilization was found to be 42.73% (955, CI 27.65%, 57.80%) based on the pooled evidence obtained from ten primary studies [14–23]. The finding of this study is higher than studies conducted in Ghana [28] and Uganda [29]. The variation could be explained by differences in socio-demographic factors and health policies among nations. In Ethiopia, there are trained female health workers known as Health Extension Workers who are dedicated to improving the health of mothers and children at the community level. These workers are primarily supervised and employed by the Ministry of Health in the country [13].

The subgroup analysis of ICCM utilization indicated that there was a significant difference between the study conducted at the community level [16, 17, 22, 23] and facility level [18, 20, 21] with a test of group difference Q _b_ [1] value of 9.61 and p-value of < 0.001. The level of ICCM was 53.87% (95%, CI 40.66 -67.09%) among studies conducted at the community level while the level of ICCM utilization was found to be 25.73% (95%, CI 13.81, 37.64%) among studies conducted at facility level. On the other hand, there was no significant difference in the subgroup analysis based on sample size. The level of ICCM utilization was found to be 43.64% (95%, CI 25.36%, 61.01%) and 41.73% (95%, CI 17.60%, 66.01%) among studies with sample size < 580 [14–18, 20] and ≥ 580 [14, 15, 19, 21].

Parents who had awareness about signs of illness were 2.77 times more likely to utilize ICCM service compared to their counterparts (OR = 2.77, 95%, CI 2.06, 3.74). Similarly, parents who have awareness about the availability of ICCM service were 3.64 times more likely to use ICCM service (OR = 3.64, 95%, CI 2.16, 6.14) compared to their counterparts. The finding of this systematic review and meta-analysis is comparable with a study conducted in Uganda which reported increased utilization of ICCM services among parents who had awareness compared to counterparts [29]. The findings of our systematic review and meta-analysis suggested that there is a need to create community mobilization and demand generation since women with insufficient awareness about the availability of ICCM services were less likely to utilize the services [30].

The odds of ICCM utilization were found to be 3.14 times higher among parents of under-five children who had high perceived severity of diseases compared to counterparts (OR = 3.14, 95%, CI 2.33, 4.23). The finding of this review was supported by a systematic review and meta-analysis conducted to assess the level of caregivers’ healthcare-seeking behavior for diarrhea, fever, and respiratory tract infections among children in Ethiopia [31]. Similar to our study, a survey conducted in various regions of Ethiopia revealed that the primary reason (42.2%) for not seeking care for a sick child was parents believing that “the child will get better“ [32]. The finding of this systematic review and meta-analysis suggests the importance of implementing targeted social behavior change communication for parents, focusing on the implications of common childhood illnesses and the benefits of early health seeking for a sick child.

Parents with a secondary education or higher were 2.57 times more likely to utilize ICCM for their sick children compared to those with lower levels of education (OR = 2.57, 95% CI 1.39, 4.77). This could be attributed to the fact that individuals with a secondary education or above are more likely to be knowledgeable about common childhood illnesses and ICCM services compared to their counterparts. This finding is consistent with a study conducted in Uganda, which found a positive correlation between knowledge levels and the utilization of ICCM services [29]. A study conducted about women’s literacy and child mortality in Southeast Asian countries indicated that women’s literacy had a significant impact on child mortality rate [33]. Enhancing women’s education is essential for improving the utilization of ICCM services for common childhood illnesses in low- and middle-income countries such as Ethiopia.

In this review, parents who lived within 30 min distance of the health post were 3.93 times more likely to use ICCM services compared to their counterparts (OR = 3.93, 95%, CI 2.30, 6.74). A similar finding was reported in a study conducted in Malawi where moderate access to ICCM service was found to increase the health-seeking behavior towards ICCM services [34]. Like ways, a study conducted in Zambia disclosed that parents with access to ICCM services were more likely to utilize the ervices compared to their counterparts [35]. The results of this systematic review and meta-analysis are further supported by a study carried out in Uganda, which found that parents living within a one-kilometer distance showed higher utilization of ICCM services compared to their counterparts [29]. A report by the World Health Organization (WHO) and the United Nations International Children’s Emergency Fund (UNICEF) reported that poor and disadvantaged children without access to facility-based case management are at greater risk of morbidity and mortality [1]. Improving access to ICCM services as close as possible to the community is essential for enhancing service utilization.

### Limitations of the study

This systematic review and meta-analysis has some limitations. The review was based on a limited number of available studies, which could potentially impact the quality of the findings. Additionally, we were unable to find studies specifically focusing on ICCM in the Northern Regions of Ethiopia, which may hinder the generalizability of the results.

## Conclusion

The utilization of ICCM was found to be low in Ethiopia. Factors such as parents’ awareness about the illness, knowledge of ICCM services, perceived severity of the disease, having a secondary/above level of education, and living within 30 min distance to the health post were significantly associated with the utilization of ICCM. Therefore, it is crucial to focus on creating awareness and improving access to high-quality ICCM services to reduce child morbidity and mortality from preventable causes.

## Electronic supplementary material

Below is the link to the electronic supplementary material.

Supplementary file 1: PRISMA checklist for reporting systematic review.

Suppementary file 2: Quality assessment of articles included in the meta-analysis of integrated community casemanagement of childhood illnesses using the Newcastle Ottawa scale (NOS).

## Data Availability

The data used in this study are available within the manuscript and its supporting information.

## Abbreviations

EDHS: Ethiopia Demographic and Health Survey
ICCM: Integrated Community Case management of childhood illnesses
NOS: Newcastle-Ottawa Quality Assessment Scale
OR: Odds Ratio
