# Supplementary material for: Utilization of integrated community-based case management of childhood illness and associated factors in Ethiopia: a systematic review and meta-analysis
Source: Ital J Pediatr. 2024 Jul 30;50:137. doi: 10.1186/s13052-024-01702-0 (PMC11389298; doi:10.1186/s13052-024-01702-0)
Supplement: Supplementary file 2 — Suppementary file 2: Quality assessment of articles included in the meta-analysis of integrated community casemanagement of childhood illnesses using the Newcastle Ottawa scale (NOS). [file 13052_2024_1702_MOESM2_ESM.docx]

Supplementary file 2: Quality assessment of articles included in the meta-analysis of integrated community case management of childhood illnesses using the Newcastle Ottawa scale (NOS)

| Studies | Selection | | | | Comparability | Outcome | | Total score |
| --- | --- | --- | --- | --- | --- | --- | --- | --- |
|  | Representativeness  (1) | Sample size  (1) | Non-respondents  (1) | Ascertainment of the exposure (risk factor)  (2) | The subjects in different outcome groups are comparable, based on the study design or analysis. Confounding factors are controlled (2) | Assessment of the outcome  (2) | Statistical test  (1) |  |
| Berhanu A. et al, 2020 (1) | * | * | * | ** | * | * | * | 8 |
| Debel N. 2022 (2) | * | * | * | * | * | * | * | 7 |
| Samuel S. et al 2021 (3) | * | * | * | ** | * | * | * | 8 |
| Yeheyis T. et al 2021 (4) | * | * | * | ** | * | * | * | 8 |
| Yohannes S. et al 2021 (5) | * | * | * | * | * | * | * | 7 |
| Salgedo WB. et al 2020 (6) | * | * | * | * | * | * | * | 7 |
| Gorfu MB. et al (preprint) 2014 (7) | * | * | * | * | * | * | * | 7 |
| Rikiba R. et al 2023 (8) | * | * | * | ** | * | ** | * | 9 |
| Kassa EA. et al, 2018 (9) | * | * | * | * | * | ** | * | 8 |
| Bellete M. et al 2021 (10) | * | * | * | ** | * | ** | * | 9 |

1. Berhanu A, Alemayehu M, Daka K, Binu W, Suleiman M. Utilization of Integrated Community Case Management of Childhood Illnesses at Health Posts in Southern Ethiopia. Pediatric Health Med Ther. 2020;11:459-67.

2. Debel LN, Nigusso FT. Integrated Community Case Management Utilization Status and Associated Factors Among Caretakers of Sick Children Under the Age of 5 Years in West Shewa, Ethiopia. Front Public Health. 2022;10:929764.

3. Samuel S, Arba A. Utilization of Integrated Community Case Management Service and Associated Factors Among Mothers/Caregivers Who Have Sick Eligible Children in Southern Ethiopia. Risk Manag Healthc Policy. 2021;14:431-8.

4. Yeheyis T, Lemma K, Nuramo A, Musema M, Dolmolo A, Aynalem A, et al. Level of modern health-seeking behavior for common childhood illnesses and its associated factors among mothers of under-five children in southern Ethiopia: A community based study. Heliyon. 2023;9(9):e20121.

5. Yohannes S, Habtu Y, Abreham B, Ayele M. Utilization of Integrated Community Case Management and Its Factors inSouthern Ethiopia: Facility Based-Cross-Sectional Study. Hindawi, dvances in Public Health. 2021;2021:8 pages

6. Salgedo W, Babulo S, Weldemarium T. Outcomes and associated factors of integrated community case management of childhood illnesses in rural districts of Dawro Zone, Southwest Ethiopia. . Primary Health Care, Open access 2020;10(2):340.

7. GORFU M. FACTORS AFFECTING UTILIZATION OF INTEGRATED COMMUNITY CASE MANAGEMENT OF COMMON CHILDHOOD ILLNESSES IN AGARFA WOREDA, OROMIYA REGIONAL STATE, ETHIOPIA

Uiversity of South Africa 2014.

8. Rikiba R, Regas A. Utilization of Integrated Community Case Management of Common Childhood Illness and Associated Factors among Mothers/Caregivers in Wonsho District, Sidaama Region: Ethiopia. International Journal of Pediatric Research. 2023;9(1).

9. Kassa EA, Handiso TB, Admassu B, Nigussie A. Utilization of integrated community case management service of childhood illness (ICCM) and associated factors among under-five children in Shashogo district, Hadiya zone, south Ethiopia. SAGE Open Med. 2022;10:20503121221097643.

10. Bellete M, Boke MM, Yenit MK. Child Caregiver's healthcare seeking behavior and its determinants for common childhood illnesses in Addis Ababa, Ethiopia: a community-based study. Ital J Pediatr. 2021;47(1):99.
